# Formal String Instrument Training in a Class Setting Enhances Cognitive and Sensorimotor Development of Primary School Children

**DOI:** 10.3389/fnins.2020.00567

**Published:** 2020-06-16

**Authors:** Clara E. James, Sascha Zuber, Elise Dupuis-Lozeron, Laura Abdili, Diane Gervaise, Matthias Kliegel

**Affiliations:** ^1^Geneva School of Health Sciences, HES-SO University of Applied Sciences and Arts Western Switzerland, Geneva, Switzerland; ^2^Department of Psychology, University of Geneva, Geneva, Switzerland; ^3^Center for the Interdisciplinary Study of Gerontology and Vulnerability, University of Geneva, Geneva, Switzerland; ^4^Swiss National Centre of Competences in Research LIVES–Overcoming Vulnerability: Life Course Perspectives (NCCR Lives), Université de Lausanne, Lausanne, Switzerland; ^5^Clinical Research Centre and Division of Clinical Epidemiology, Geneva University Hospitals and Faculty of Medicine, University of Geneva, Geneva, Switzerland

**Keywords:** musical instrumental practice, group setting, cluster randomized controlled trial, multiple transfer effects, cognitive, sensorimotor, matrix reasoning, string instruments

## Abstract

This cluster randomized controlled trial provides evidence that focused musical instrumental practice, in comparison to traditional sensitization to music, provokes multiple transfer effects in the cognitive and sensorimotor domain. Over the last 2 years of primary school (10–12 years old), 69 children received group music instruction by professional musicians twice a week as part of the regular school curriculum. The intervention group learned to play string instruments, whereas the control group (i.e., peers in parallel classes) was sensitized to music via listening, theory and some practice. Broad benefits manifested in the intervention group as compared to the control group for working memory, attention, processing speed, cognitive flexibility, matrix reasoning, sensorimotor hand function, and bimanual coordination Apparently, learning to play a complex instrument in a dynamic group setting impacts development much stronger than classical sensitization to music. Our results therefore highlight the added value of intensive musical instrumental training in a group setting within the school curriculum. These results encourage general implementation of such training in public primary schools, thus better preparing children for secondary school and for daily living activities.

## Introduction

Practicing a complex instrument regularly and actively over extended periods of time may provoke positive transfer effects on basic and higher order cognition as well as on sensorimotor skills in children (Costa-Giomi, 2004; Schellenberg, 2004, 2006; Palac and Sogin, 2005; Schlaug et al., 2005; Moreno et al., 2011; Roden et al., 2012; Bergman Nutley et al., 2014; Seither-Preisler et al., 2014; Tierney et al., 2015; Martins et al., 2018). Some studies even suggest long-term effects of musical practice during childhood (Schellenberg, 2006; Hanna-Pladdy and Mackay, 2011; White-Schwoch et al., 2013; Balbag et al., 2014; Moreno et al., 2015). In children suffering from dyslexia and attention deficit hyperactivity disorder (ADHD), observed interhemispheric asynchronies in auditory cortices, associated with their deficits, were less prominent in those practicing a musical instrument (Seither-Preisler et al., 2014; Serrallach et al., 2016). Several authors note that besides practice effects, innate talent and contextual elements such as the type of pedagogic intervention and parental support may also strongly impact cognitive and cerebral benefits (Corrigall et al., 2013; Seither-Preisler et al., 2014; Corrigall and Schellenberg, 2015; Sala and Gobet, 2017).

However, as summarized by Dumont et al.’s (2017) recent review, the available literature is largely inconclusive because of the lack of randomized controlled trials (RCTs) and active control groups. The authors also observed a great heterogeneity across the different studies, with regard to group size, intensity and nature of the music regimens. Of the 46 studies on music interventions that the Dumont review comprised, only two used an RCT design. Neither of these two studies, lasting 6 months and 6 weeks, respectively, could show cognitive benefits in the music groups compared to art classes (Mehr et al., 2013; Flaugnacco et al., 2015).

Specifically concerning group training, Rickard et al. (2012) investigated school-based instruction in young adolescents over 5 to 6 months in a pseudo-randomized study. No convincing developmental benefits of music lessons in a class setting manifested in comparison to the control groups that received drama and art classes. The proposed music trainings (Rickard et al., 2012) involved conscious listening and introduction to basic musical concepts, playing and improvising on different instruments and learning different musical notations, but did not involve focused musical instrumental training. Another group setting study (Degé et al., 2011) compared children in similar age groups as in the current study (9–11 years old) after 2 years of intensive school music training, with a passive control group. Participants were not assigned randomly to the music groups. The authors found evidence for enhanced short-term auditory and visual memory in the music groups. Their musical regimen did involve playing a particular instrument. Slater et al. (2014), in a pseudo randomized group setting, observed a catch-up in reading performance in low-income children after 1 year of music training comprising focused instrumental training as well as musical theoretical education. However, they used a passive control group and only children who desired to participate were included in the music group, inducing a motivational bias.

In a cross-sectional study, Jaschke et al. (2018a) observed in 6-year-old children that extracurricular exposure to a musically enriched environment (listening) did not provoke significant relationships with cognitive function, although a trend with verbal intelligence appeared.

Rose et al. (2015) adopted an original approach, using a mixed design to appraise on a continuum the multifactorial characteristics of musicality in 38 young children (7–9 years old) after learning a musical instrument for over 1 year. This study was not an RCT, and the extra classes could be chosen, which represents a motivational bias. Group classification (more or less musical training) depended on the amount of received musical training. The results suggested there were some advantages to music lessons on hand-eye coordination and non-verbal reasoning.

A longitudinal interventional study spanning over 1 year within the school curriculum compared 128 young adolescents (mean age ∼11 years at start), all receiving 2 h of standard music training. Around half of them received another 2 h of intensive music training (music curriculum) comprising 1 h of private instrumental tuition, and the other half 2 h of art and science classes (standard curriculum; Carioti et al., 2019). This study was not an RCT, and the extra classes could be chosen. Carioti et al. (2019) did not control for previous music experience: ∼40% of the music curriculum children received previous music training against ∼75% of the groups that received only standard music training plus art classes. Finally, the children who received previous music lessons came from families with a higher cultural background. Despite this imbalanced experimental plan, the music curriculum children showed cognitive and visuo-spatial advantages after 1 year.

Finally a recent longitudinal study over 2.5 years (Jaschke et al., 2018b) compared young primary school children (mean age 6.4 years at the beginning of the interventions) following music and art classes one or two times per week, in addition to the regular school curriculum. Initially, the study comprised three experimental groups -a music, a visual arts and a passive control group- applying cluster randomization per school. The music intervention involved acquiring basic music knowledge and listening, and also encouraged the children to play instruments, but no focused instrumental training was targeted over the full period of the intervention. Therefore, this training represents in our point of view an enriched sensitization to music. Additionally, Jaschke et al. (2018b) added a fourth music group *post hoc* to the study, that was not part of the randomization process and that consisted of children who received extracurricular private musical instrumental lessons prior to and during the school music intervention to which they also participated. Inhibition, planning and verbal IQ improved in both music groups as compared to the art and passive control groups. No differences were found between the two music groups. In our point of view, the results are to some extent contaminated by adding a *post hoc* group of children receiving private lessons outside the randomization process.

From all these studies, we conclude that it seems predominantly focused instrumental practice (i.e., learning to play a complex musical instrument over an extended period of time) that constitutes the main driving force for far transfer to basic and higher order cognitive processing and sensorimotor skills. However, so far, this has not been investigated systematically within a long-term RCT with an active control group, in an intracurricular and thus a natural class setting, involving children of different backgrounds and using a large behavioral battery covering distinct developmental domains.

Available evidence of the beneficial effects of musical practice on a child’s cognitive development predominantly concerns children of parents with a high economic and educational background (Corrigall and Schellenberg, 2015) and typically results from private lessons. Additionally, most of the time, the child is interested in learning a musical instrument, thus inducing a motivational bias (Corrigall et al., 2013). Evaluation of beneficial transfer effects is restrained to a limited number of capacities or skills in general, and RCTs with active control groups are scarce.

Here, we compared children who intensively practiced different string instruments in a class setting within a specific Orchestra in Class (OC) program to peers in parallel classes who received the same amount of musical instruction, also within an entire class, but who lacked focused training on a complex musical instrument. Entire existing classes were assigned randomly to the OC and the control programs.

The study took place in public primary schools in popular (low-income) neighborhoods in the Geneva area avoiding confounding the effects of music education with the effects of socioeconomic background.

We anticipated that cognitive functions strongly involved in musical practice such as working memory, attention, information processing, cognitive flexibility, and abstract reasoning, as well as fine sensorimotor function would provoke enhanced positive transfer effects in the OC group as compared to the control group. Results showing transfer of fine sensorimotor training to other sensorimotor tasks following musical training are rather scarce, although a few studies have reported the advantages of musical practice to other sensorimotor actions outside the domain of music (Palac and Sogin, 2005; Martins et al., 2018).

## Materials and Methods

### Participants

Sixty-nine primary schoolchildren participated in the study (*M* at baseline = 10.18 years; *SD* = 0.31; 41 girls). Using a sociodemographic questionnaire, we checked for developmental and neurological disorders, hearing deficits or other important health issues that did not occur in this population.

We admit that the lack of socio-economic information on the parents is a weak spot of the study. One of the school principals did not allow the distribution of our questionnaire on the parents’ socioeconomic background for ethical reasons. We recruited the children at the establishments where the OC program was integrated in the regular curriculum in French-speaking Switzerland, in neighboring public schools, in a popular (low-income) neighborhood, therefore hosting children of varying ethnic and of relatively low socioeconomic backgrounds. There are many immigrants (including refugees) in Geneva who cannot pay high rents, and therefore several different ethnic groups frequent the schools where our study took place. More than 40% of the children were bilingual, and sixteen different second languages were reported. Two nearby establishments in the same Geneva suburb, both consisting of two different sub-schools in different buildings, participated in the study. These establishments collaborate intimately, for instance by exchanging pupils to compose balanced classes over the years.

At baseline, before the interventions, the children had almost finished their sixth year of elementary school, one of eight consecutive years covering ages between 4 and 12 years approximately. We excluded any children who had followed regular or protocolled music practice outside the school curriculum before the study. Seven children were left-handed, three in the control group and four in the OC group (see Supplementary Table S1). We integrated handedness in the linear mixed models, controlling for this factor (see the section “Linear Mixed Models”).

#### Consent

The children and their parents or caregivers signed an informed consent to participate in the study. We informed them that the study would be performed by the Faculty of Psychology and Educational Sciences of the University of Geneva. We provided assurance that all data would remain strictly confidential and that only the project leader would have access to the link between the codes assigned to the children and their names.

#### Ethics

The ethics commission of the Faculty of Psychology and Educational Sciences of the University of Geneva approved the protocol in agreement with the ethical standards of the Helsinki declaration.

#### Blinding and Confidentiality

As this was a “field study” it was not feasible to blind children and experimenters for group affiliation because the courses took place within the regular school curriculum. The experimenters knew the two groups existed, but we did not inform them about the hypotheses. We accorded each child a number code in the first test passage that protected his/her identity. Only the principal investigator could later access the files that linked each child with his/her code and group. The statistician who performed the final analyses was ignorant to the subjects’ identity and the classes they belonged to.

### Musical Interventions

The assignment of whole classes to either the OC group or the control group was random (cluster randomization; Mazor et al., 2007). The allocation of entire classes (cluster randomization) to either group resulted from administrative considerations (availability of the music room and of the music teachers). Motivation from the children or the titular schoolteacher of a class did not play a role in the assignments. The study’s investigators were not involved in this process either. Therefore, the assignment of the classes to the intervention group or the control group can be considered random.

The most important advantage of a cluster randomization in our context is that the intervention was tested under representative natural conditions (Mazor et al., 2007), as the Geneva OC program provides the group music lessons within the regular school curriculum in existing classes.

In the Geneva canton, children are assigned to schools based on the neighborhood, and not the parents’ choice, and as the neighborhood was popular (low-income), it may be assumed that socioeconomic level was more or less equally distributed in the different classes.

**FIGURE 1:**
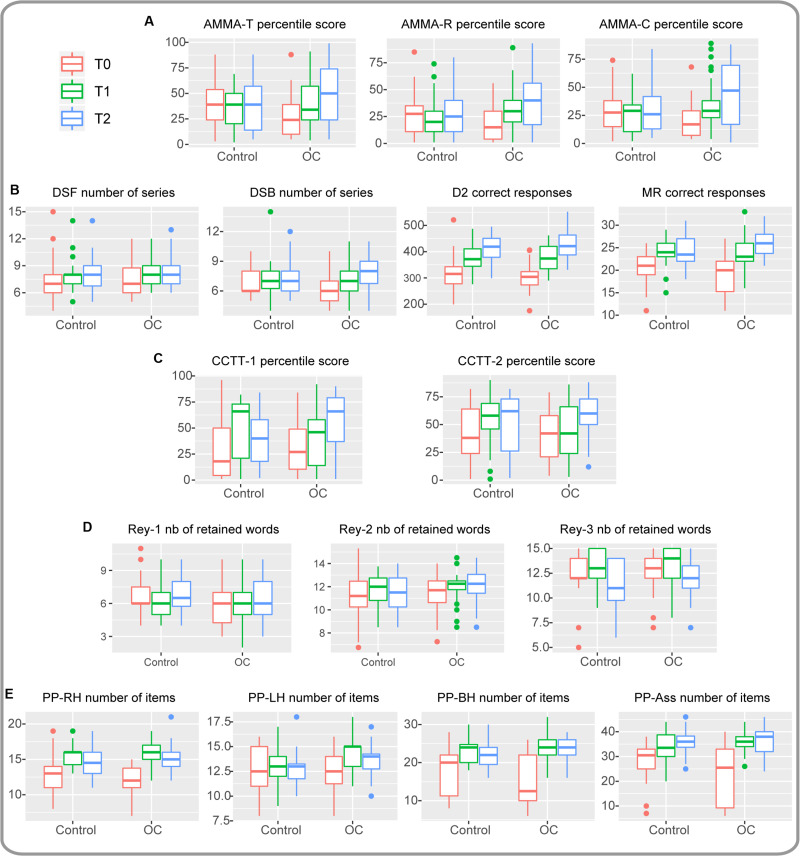
Raw data are represented as boxplots around the median, with lower and upper hinges corresponding to the first and third quartiles. The upper (respectively lower) whisker extends from the hinge to the largest (respectively smallest) value no further than 1.5*IQR (interquartile range) from the hinge. Outliers are represented by dots. T0: baseline, T1 after one year, T2 after two years. **(A)** AMMA-T, AMMA-R and AMMA-C percentile scores. **(B)** DSF, DSB, D2, and MR scores. **(C)** CCTT-1 and CCTT-2 percentile scores. **(D)** Rey-1, Rey-2, and Rey-3 scores. **(E)** PP-RH, PP-LH, PP-BH, and PP-Ass scores (see Table 1 for an explanation of the acronyms and involved abilities of all tests items).

As a consequence of the cluster randomization, baseline performances differed between the intervention group and the control group (see Figure 1 and Supplementary Table S1). This represented a bias as we wanted to evaluate the development/progress, independent of the baseline performance. Therefore, we controlled for baseline differences in the linear mixed models (see the section “Linear Mixed Models”). Finally, we excluded all children who followed music lessons prior to the interventions from the study in order to exclude bias that could occur if, for instance several children in either group would have followed regular musical lessons before the interventions.

Even in the case of a classical RCT it is recommended adjusting the analyses on variables that may influence the dependent variable. For example, the guidelines of the European Medicines Agency mention that “Baseline covariates impact the outcome in many clinical trials. Adjustment for such covariate(s) generally improves the efficiency of the analysis and avoids conditional bias from chance covariate imbalance” (European Medicines Agency [EMA] and Committee for Medicinal Products for Human Use [CHMP], 2013).

The intervention group (OC) comprised 34 children (two classes), and the control group included 35 (two classes). All interventions were only provided in a full class within the school curriculum, and children received either OC or active control interventions but never both. Full classes comprised ∼20 children (school classes are small in the Geneva canton). As we excluded from our analyses children who followed protocolled music lessons before the interventions (*n* = 5, two in the OC classes and three in the control classes), some families moved away, and other children had to repeat classes, our analyses were performed on four groups of seventeen or eighteen pupils, two in the OC program and two in the control program. OC groups always received their training in a full class or “orchestra” setting, with all four different instruments present. No individual lessons or lessons in smaller groups were provided in either group.

#### Orchestra in Class (OC) Group

Children received OC courses within a whole class two times per week for 45 min and within their own school during the last 2 years of primary school. Two teachers, one for the higher string instruments (violin, viola) and one for the lower ones (cello, double bass) were present at all courses. First the children were assigned their instrument (violin, viola, cello or double bass). In the first lessons, the children became acquainted with all the instruments: The teachers played on them, and then the children could try them out themselves. Next they listed on a form two instruments in order of preference and added a small argumentation (f.i. “I absolutely do not want to play the violin, because …”). In principle, first or second choices could be respected. The teachers instructed the few children who were not satisfied with their assigned instrument to work out a solution together and then redistributed the instruments among them in such a way that the highest possible level of satisfaction was obtained for each child.

At the beginning playing involved bowing open strings smoothly or “legato” (without using the fingers of the left hand) and using pizzicato (plucking the strings with the right hand), in order to familiarize the child with the instrument. Then, the children progressively used their left-hand fingers to stop the strings: first while playing pizzicato, in order to concentrate on the fingers of the left hand, and later on in combination with more and more diversely articulated use of the bow. Meanwhile, rhythms evolved from very simple and regular to more complicated and irregular ones. After 3 months, the children could take their instruments home and were encouraged to practice on a voluntary basis. After 1 year, the average child could play on all strings with all four fingers of the left hand (the thumb stabilizes the neck of the instrument) and could use varied bowings. Score reading and basic solfège were gently initiated during the first year but were largely applied in the second year.

To develop auditory skills, the children learned to play musical pieces first by imitation (“trial and error”) – that is, repeating elements of each piece after the teachers played it. Progressively the elements were put together. They also sang the pieces. Additionally, the teachers played discrimination games with the children: “are these two patterns the same?,” as well as imitation games, that involved repeating short patterns after the teacher with closed eyes. The children learned solfège retroactively: first, playing pieces/patterns and then linking the sound to the musical notation that the teachers explained. So, auditory perception always preceded note reading. At all times, from the very beginning, ensemble playing remained a priority, and the teachers constantly made the child aware of being part of a whole within a polyphony.

Learning followed three paths. This included imitating the teacher and reading the score later on. Finally, emulation among students was another important vehicle of learning. Small concerts and events stimulated the children and gave purpose to their learning, including two study weekends with a final concert in front of the families.

The OC teachers in this study, all professional musicians with a master’s degree, followed a 10-day specific training to teach OC at the ITEMM in France (Institut Technologique Européen des Métiers de la Musique/European Technological Institute for Music Professions). The method is based on the direct transmission from listening to playing, but note reading is integrated on a posthoc basis: first learning how a piece of music sounds, then playing it on the instrument and, finally understanding the notation. All exercises are performed in a group setting, involving a whole class.

#### Sensitization to Music

In the Geneva canton all children in this age group attending public schools, receive 45 min of musical education twice per week, and the same was true for the control group here during the last 2 years of primary school. The latter education is best described as “sensitization to music” and involves listening actively, -that is, learning to recognize instruments and themes, learning some theory, singing together and playing small percussive instruments or the recorder. The proportion of the musical activities in the active control classes was highest for singing, followed by listening, learning some history (involving homework) and incidental use of simple instruments. The teachers were professional musicians who received training to provide musical education in a school setting. The children, like in the OC group, also participated in class performances for the parents.

### Procedure

Research assistants, master students of the Psychology Department of the Geneva University tested all children individually within the school that the child attended. These research assistants tested the children three times: at baseline, before the lessons started (T0), after 1 year (T1) and after 2 years (T2). The experimenters encouraged each child to ask questions and emphasized that he/she may ask for a break at any time. All experimenters were well trained (two 3-h sessions) to pass the tests correctly and uniformly beforehand.

The tests were administered in pseudo-randomized order, in a time window of approximately 2 h altogether. The total testing time was one hour and a half, separated by several breaks. The children received a small gift at the end of each session.

### Materials

Table 1 presents all types of tests, level of transfer, measured variables, acronyms of the tests and involved abilities. We chose all tests assuming influence by musical instrumental training and musical capacity on the measured abilities: musical aptitude; auditory short-term and working memory; attention; processing speed; cognitive flexibility, the latter specifically solicited in a group setting (Bugos and Mostafa, 2011; Bergman Nutley et al., 2014; Roden et al., 2014); abstract thinking (underpinned by working memory; Cowan, 2014); verbal memory (supposed to be enhanced by music training (Ho et al., 2003; Roden et al., 2012; Jaschke et al., 2018b), and, given the complexity of playing a string instrument, sensorimotor hand function and bimanual coordination (Palac and Sogin, 2005).

**TABLE 1:** Types of tests, level of transfer, measures, acronyms, and involved abilities of all items of the test battery.

Type of test	Measure	Acronym	Involved ability
**Musicality;** close transfer			
Advanced Measures of Music Audiation	Tonal score	AMMA-T	Tonal processing
	Rhythmic score	AMMA-R	Rhythmic processing
	Composite score	AMMA-C	Tonal and rhythmic processing
**Cognitive Function;** far transfer			
Digit Span	Digit span forward	DSF	Short-term memory
	Digit span backward	DSB	Working memory
Test of Attention	D2	D2	Selective attention (visual) and processing speed
Matrix Reasoning		MR	Fluid intelligence/abstract reasoning
Children’s Color Trails Test	Subtest 1	CCTT-1	Processing speed (visual)
	Subtest 2	CCTT-2	Processing speed (visual) and cognitive flexibility
Rey Auditory Verbal Learning Test	Recall	Rey-1	Verbal short-term memory (STM)
		Rey-2	Verbal learning
		Rey-3	Verbal long-term memory (LTM)
**Sensorimotor Skills;** intermediate transfer			
Purdue Pegboard	Right hand	PP-RH	Gross dexterity right hand
	Left hand	PP-LH	Gross dexterity left hand
	Both hands	P-BH	Gross dexterity of both hands and bimanual coordination
	Assembly	PP-Ass	Bimanual coordination and fine finger dexterity

#### Music Audiation

As simply possessing good discriminatory skills for pitch and rhythm is not sufficient to evaluate musical aptitude, we administered the “Advanced Measures of Music Audiation” (AMMA; Gordon, 1989). This test requires the capacity to group individual notes into “Gestalts” and to form expectancies thus evaluating “auditory structuring” (Karma, 2007). The AMMA test does not require any prior musical knowledge or skills and is suited to evaluate musical aptitude in preadolescents up to professional musicians (Grades 7 to Adult). The test encompasses thirty trials consisting of pairs of musical melodies presented over headphones via the computer. For each pair, the children judged whether the melodies were identical or different, and if they considered the two melodies of the pair to be different, they had to indicate whether the difference was melodic or rhythmic. Tonal and rhythmic differences never occurred together. Among the 30 pairs, 10 pairs are identical, 10 are melodically different and 10 are rhythmically different. The first phrase always contains exactly the same number of notes as the second. Scoring is divided into a tonal sub-score, a rhythmic sub-score and a composite score that is a combination of both tonal and rhythmic scores. The performance on this test represents an interaction between innate musical potential and exposure to musical environments. Because the scoring system penalizes errors, we advised the children not to respond if they were not sure. To prevent errors, the experimenters filled out the answer sheet that contained four columns: identical; melodic difference; rhythmic difference; and “I don’t know.” After explaining the concepts of melody and rhythm in a plastic way, the children passed three training trials that were discussed with them to ensure that they understood the instructions. We computed a tonal, a rhythmic and a composite standard score, the latter composed of both tonal and rhythmic scores, according to the AMMA manual (Gordon, 1989), thus applying a subtraction of points for wrong answers. From these standard scores we inferred percentile rank scores (category of high school students) according to the AMMA manual (Gordon, 1989), which we used for the analyses.

We chose the AMMA test instead of the IMMA (Intermediate Measures of Musical Audiation; ages seven to eleven) because the children would be 12 years or older old at T2, and we wanted to use the same test in order to allow direct comparison. Raw scores were low at T0, but at T2 the OC children showed percentile scores with a median around 50% for the composite score (see Figure 1A) in the category “high school students.” The AMMA is suited for junior high students^1^. Researchers have repeatedly reported that this test is capable of measuring musical aptitude distinctively in musicians and non-musicians, also from different cultures (Ruthsatz et al., 2008; Kołodziejski, 2010; Hanson, 2019).

#### Digit Span Forward and Backward

All children passed the “digit span” subtest of the Wechsler Intelligence Scale for Children - Revised (WISC-R; Wechsler, 2005). In this test the participant listens to series of digits with increasing length and must repeat them orally: in direct order in the digit-span forward (DSF) task and in reverse order in the digit-span backward (DSB) task. To ensure a regular time course (1 s per digit) and identical pronunciation of the presented material for all participants, we prerecorded the spoken series. DSF and DSB assess distinct but interdependent cognitive functions (Grégoire, 2009). DSF evaluates essentially short-term auditory memory, whereas DSB principally evaluates the ability to manipulate verbal information while temporarily stored, thus auditory working memory capacity. The research assistants presented two series of digits (one for each task), progressively increasing in length and thus in difficulty. The children first performed the DSF (span size from two up to nine) then the DSB task (span size from two up to eight). The task was interrupted if the child made two successive mistakes with the same number of digits (i.e., at the same level of difficulty). Each correct answer counted for one point.

#### D2 Test of Attention

To assess the children’s selective visual attention, sustained attention and visual scanning speed (processing speed), we administered the D2 test of attention (Brickenkamp and Zillmer, 1998). Stimuli consisted of the letters d or p, accompanied by one or two apostrophes above and/or below the letter, presented on a paper sheet with 14 rows of 47 stimuli. The participant crossed out all the d’s accompanied by exactly two apostrophes (i.e., two apostrophes above, two apostrophes below or one above and one below the d), without crossing out any of the distractors (d’s accompanied by only one apostrophe and all p’s). To familiarize the children with the task, they first performed a practice row of 22 trials. For the actual task, children started working on the first row, and were summoned to switch to the next row every 20 s. The outcome measure (D2) we used provides the total number of correctly marked items minus the number of errors and omissions.

#### Matrix Reasoning

To appraise abstract reasoning, we applied the matrices subtest of the WISC-IV (Wechsler, 2003). Since musical phrases develop over time according to musical grammar, like language (Patel, 2012), we consider that some abstract thinking is involved in producing and processing music (Jaschke et al., 2018b). The test consists of different sheets with a series of three images (e.g., three oval shapes). The child should detect the image that correctly completes the series (e.g., another oval shape) among four distractors (e.g., other shapes). Prior to the task, the children went through three practice trials to ensure they understood the instructions. For the real task, the sheets gradually increased in difficulty. The task was interrupted when the child answered four out of five consecutive sheets incorrectly. The number of correctly answered sheets constitutes the final score.

#### Children’s Color Trails Test

The Children’s Color Trails Test (CCTT; Llorente, 2003) consists of two subtests. In the CCTT-1 test we presented children a sheet with 15 circles containing the digits “1” to “15.” The child connected the digits in increasing order with a pencil as rapidly and correctly as possible. All circles with even digits were colored yellow, whereas the circles with odd numbers were colored pink. For the CCTT-2 test, children performed the same task with the following difference: For each digit (except for number “1”) two circles were presented on the sheet, one colored yellow, the other colored pink. Children were instructed that in addition to connecting the digits in increasing order, the colors of the circles would have to alternate for each digit (the pink “1” had to be connected with the yellow “2,” which had to be connected to the pink “3,” etc.). The CCTT-1 evaluates simple visual processing speed, whereas the CCTT-2 evaluates visual processing speed plus cognitive flexibility. Both subtests started with an eight-digit practice sheet for familiarization purposes. We computed standard scores as outcomes for both subtests (*M* = 100; *SD* = 15). For the analyses we used associated percentile scores.

#### Rey Auditory Verbal Learning Test

The research assistants presented a list of 15 unrelated words orally to the children five times in a row (Rey, 1964; Bean, 2011). Each time, the children repeated as many correct words from the list as possible after a short break of approximately 10 s. The words could be repeated in random order. The time limit for recollection was set at 1 min for the first trial, and for trials two to five to 1 min and 30 s. The list was read aloud first each time. The children performed trials two to five immediately after trial one. After a delay of about 50 min, the children again recited as many words as possible from the list but this time without an oral presentation beforehand. The scores represent the number of correctly repeated words for each trial. We composed the following measures: (1) the score of trial 1 (Rey-1) evaluating verbal short-term memory (STM); (2) the mean score of trials two to five (Rey-2), evaluating verbal learning; and (3) a score of delayed recall (Rey-3), evaluating verbal long-term memory (LTM). At each time point we used different lists.

#### Purdue Pegboard

The Purdue Pegboard (PP) test, administered according to the Lafayette manual (Lafayette, 1999), serves to measure manual gross and fine dexterity as well as bimanual coordination. The PP contains two parallel rows of 25 vertically oriented holes. Two cups on top of the board contain pegs (diameter 1 mm), collars and washers. After a familiarization phase, the children inserted as many pegs as possible into the holes in 30 s, from top to bottom, first with their right hand (PP-RH), then with their left hand (PP-LH) and finally with both hands simultaneously (PP-BH). PP-RH, and PP-LH evaluate gross hand dexterity, and PP-BH evaluates gross hand dexterity and also bimanual coordination. Then, again after a familiarization phase, the children performed an assembly task working with both hands together, placing as many assemblies in the holes as possible in 1 min (PP-Ass). This subtask requires bimanual coordination in combination with fine finger dexterity. One assembly consisted of a peg, a collar and two washers (four elements) to be placed into one hole in a specific order. We collected four scores, corresponding to the number of pegs placed (PP-RH, PP-LH, PP-BH) and the number of correctly inserted elements placed during the PP-Ass task.

### Missing Data

We report, depending on the test (see Supplementary Table S1 for more details), one missing value at most at T0 (AMMA and PP tests), two missing values at T1 for all tests and from five to six missing values (AMMA test) at T2.

### Retrospective Power Analysis

In order to verify the power of our study a posteriori, we performed -for two important test scores- 500 simulations, using the observed values as theoretical values for the simulations. We obtained a power of ∼70% for the backward digit span test for our sample size (i.e., we could reject the null hypothesis in ∼70% of the simulated samples where the null hypothesis was false, using the estimated values of the models’ parameters as values for these parameters under the alternative hypothesis to create the samples), and of ∼90% for the matrix reasoning test.

### Analyses

#### Linear Mixed Models

Linear mixed models are a generalization of ANOVA and linear regression for situations where measures are repeated on the same individuals. We chose linear mixed models instead of an ANOVA or ANCOVA. Linear mixed model approaches are a generalization of ANOVA type models with the advantage of being more flexible and powerful, because they can handle several levels of clustering, continuous and qualitative explanatory variables and imbalanced data. For instance, the qualitative variables of gender and manual laterality (handedness) were imbalanced between the groups, and there are different numbers of missing values depending on the group for several tests (see Supplementary Table S1). ANOVA/ANCOVA type models were developed for balanced data, in which case they provide exact inference. When this is not the case, mixed effects models are preferable (Pinheiro and Bates, 2006). Additionally, linear mixed-effects models also allow the researcher to provide estimated means and confidence intervals.

We composed three linear mixed model equations for each test in order to model the evolution of scores over time (T1 versus T2) for each child from each group, with a random intercept for each child. All models comprised the score at T0 (Score ∼ T0), age at T0 (Age_2016), gender (SEX), and handedness [LAT (for manual laterality)] to control for these factors.

**Model 1:** Time^∗^Group Interaction, to verify whether Groups evolve differently over Time:
Score ∼ T0 + SEX + Lat + Age_2016 + Time + Group + Time:Group
**Model 2:** Effect of Time and Group:
Score ∼ T0 + SEX + Lat + Age_2016 + Time + Group
**Model 3:** Effect of Time:
Score ∼ T0 + SEX + Lat + Age_2016 + Time

The lme4 and the emmeans package of the software R (3.6.0.) served to estimate the model parameters^2^, freely available at http://www.R-project.org (Bates et al., 2015).

#### Likelihood-Ratio Tests

We assessed the statistical significance of the Interaction effect Time^∗^Group and of the main effect of the factor Group using likelihood-ratio tests (Pinheiro and Bates, 2006).

In our context, a significant main effect of group indicates a significant difference between the two groups for the two time points (T1 and T2) collapsed (corrected for the score at T0). Significant interaction implies that the differences between the two groups change between T1 and T2 (corrected for the score at T0).

To do so, we compared models with and without the factor of interest (Interaction Time^∗^Group; Group). So, we tested the significance of the interaction Time^∗^Group by comparing the first and the second model with a likelihood-ratio test. In the same way we tested the effect of the group by comparing the second and third models.

This procedure resulted in values of the observed chi-square test statistic, associated *p*-values, and effect sizes (partial Rsquare/r^2^ at the level of the test and Rsquare/r^2^ at the level of the model) for Interaction Group^∗^Time and Group (see Table 2). *R*^2^ was computed using the R function r2beta of the r2glmm package. This function uses the method proposed by Edwards et al. (2008). We applied the Kenward Roger approach to approximate the denominator degrees of freedom of the F statistics of the fixed effects used in the computation of the *R*^2^.

**TABLE 2:** Results of the two likelihood tests comparing the linear mixed models (T1 vs. T2), expressed by means of observed chi-square (Chi_2) test statistics, associated *p*-value, and effect size (partial Rsquare/r^2^ at the level of the test and Rsquare/r^2^ at the level of the model, both expressed in percentage).

Measure	Likelihood test	Chi_2	*Df*	*p*-Value	Partial *R*^2^	*R*^2^_model
AMMA-T	Interaction Time*Group	0.406	1	0.524	0.1	12.5
	Group	5.028	1	**0.025**	9.4	
AMMA-R	Interaction Time*Group	0.024	1	0.877	0.1	21.7
	Group	8.013	1	**0.005**	13.1	
AMMA-C	Interaction Time*Group	0.167	1	0.683	0.0	22.4
	Group	10.368	1	**0.001**	15.8	
DSF	Interaction Time*Group	0.001	1	0.978	0.0	51.1
	Group	1.067	1	0.302	1.6	
DSB	Interaction Time*Group	3.225	1	*0.073*	4.8	39.8
	Group	4.650	1	**0.031**	7.0	
D2	Interaction Time*Group	1.392	1	0.238	2.1	64.8
	Group	4.044	1	**0.044**	5.9	
MR	Interaction Time*Group	3.001	1	*0.083*	4.4	30.5
	Group	4.571	1	**0.033**	6.7	
CCTT1	Interaction Time*Group	6.989	1	**0.008**	9.5	14.7
	Group	0.174	1	0.677	0.4	
CCTT2	Interaction Time*Group	8.528	1	**0.004**	14.1	33.6
	Group	1.074	1	0.300	0.8	
Rey-1	Interaction Time*Group	0.473	1	0.492	0.7	23.3
	Group	0.335	1	0.563	0.5	
Rey-2	Interaction Time*Group	0.335	1	0.563	0.5	23.3
	Group	2.738	1	*0.098*	4.1	
Rey-3	Interaction Time*Group	1.690	1	0.194	2.6	23.3
	Group	1.135	1	0.287	1.7	
PP-RH	Interaction Time*Group	0.302	1	0.582	0.5	34.7
	Group	7.002	1	**0.008**	10.0	
PP-LH	Interaction Time*Group	0.619	1	0.431	0.7	35.8
	Group	7.958	1	**0.005**	15.9	
PP-BH	Interaction Time*Group	0.022	1	0.882	0.0	33.3
	Group	8.892	1	**0.003**	16.0	
PP-Ass	Interaction Time*Group	0.481	1	0.488	3.3	11.0
	Group	3.994	1	**0.046**	6.9	

*Significant effects are represented in bold font. Marginal but non-significant effects (*p* = 0.05–0.1) are represented in italic font. The linear mixed models all comprised score at T0, age at T0, gender and handedness in order to control for these factors (see the section “Linear Mixed Models”).*

As we were principally interested in comparing the development of the children as a function of the two musical interventions over time, we did not investigate the main effect of Time. A significant effect of time would only indicate that children of both groups showed better results at T2 than at T1, which was expected. Moreover, whether the main effect of time was significant or not would not inform us about the potential differences between the groups, which represents our study goal.

## Results

**FIGURE 2:**
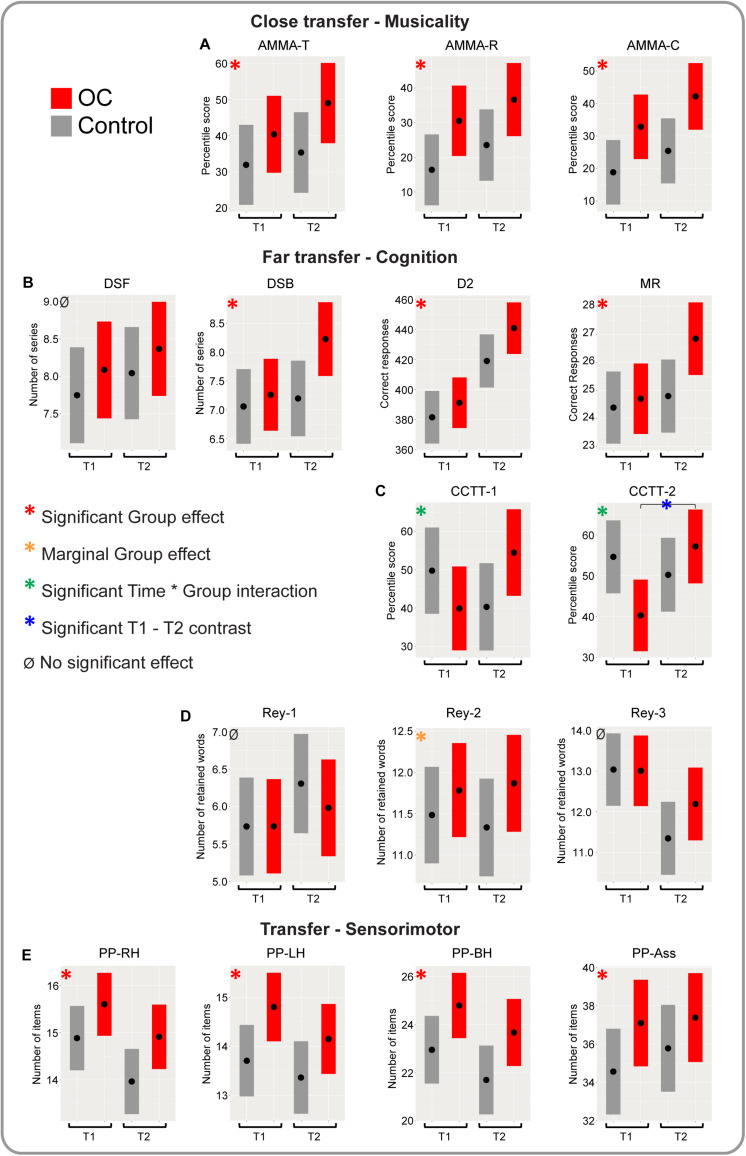
Illustration of the results provided in Table 2 for all measures. Only estimated scores assessed by means of the linear mixed models at T1 and T2 are provided in Figure 2, as the score at T0 was incorporated in the model. Results of the two likelihood tests comparing the linear mixed models (T1 vs. T2), are represented by red asterisks depicting a main significant effect of group (T1 and T2 collapsed) and by orange asterisks depicting a marginal (but not significant) main effect of group (*p* = 0.05–0.1). Significant interaction effects are depicted by green asterisks and represent a significant Time*Group interaction. Blue asterisks depict a significant Tukey corrected contrast OC group vs. control group, which was only computed at T2 for CCTT-1 and CCTT-2. **(A)** AMMA-T, AMMA-R and AMMA-C estimated percentile scores. **(B)** DSF, DSB, D2, and MR estimated scores. **(C)** CCTT-1 and CCTT-2 estimated percentile scores. **(D)** Rey-1, Rey-2, and Rey-3 estimated scores. **(E)** PP-RH, PP-LH, PP-BH, and PP-Ass estimated scores (see Table 1 for an explanation of the acronyms and involved abilities of all tests items).

All types of tests, measured variables, acronyms of the tests and involved abilities are resumed in Table 1. Supplementary Table S1 provides mean descriptive data per group. Raw average data can be visualized by means of boxplots of all variables and at all three time points (T0, T1 and T2) in both groups in Figure 1. The final statistical results are represented in Table 2 and illustrated in Figure 2. These final outcomes are the result of two-by-two comparisons of three different linear mixed models using likelihood-ratio tests for each comparison (see the section “Likelihood-Ratio Tests”). We controlled for the scores at T0, age, gender and handedness by incorporating their values within the linear mixed models. The “Materials and Methods” section describes the syntax of the three linear mixed models and their comparisons by means of likelihood-ratio tests. The outcomes from the two likelihood-ratio tests answer the following two questions: whether effects of (1) Interaction Time^∗^Group and or (2) Group (OC vs. control) were significant. The interaction test responds to the question whether the two groups developed differently over time. In Figure 2, we provided exclusively the estimated scores assessed by means of the linear mixed models at T1 and T2, because the score at T0 was incorporated into the models in order to correct for differences at baseline between the groups, as assignment of entire classes to the OC group or control group was random (cluster randomization).

For the sake of brevity and transparency, statistical results can be found mainly in Table 2 and as little as possible in the text. We will report on the results in detail below as a function of transfer: near transfer (music processing), far transfer (cognition), and sensorimotor transfer.

### Near Transfer

All children passed the “Advanced Measures of Music Audiation” of Gordon (AMMA; Gordon, 1989). In the AMMA test subjects compare melodies and judge whether they are identical or whether melodical/tonal or rhythmic differences occur. The OC group showed significantly enhanced percentile scores compared to the control group, with T1 and T2 collapsed (from now on “main effect of group”), for both the tonal subtest (AMMA-T) and the rhythmic subtest (AMMA-R) and thus also for the composite test (AMMA-C), which is a combination of both tonal and rhythmic scores so as to provide an overall musicality score (see Table 2 and Figure 2A). A progression in the scores occurred between T1 and T2 (corrected for T0) in both groups. Interaction Time^∗^Group was not significant, which means that the evolution of the scores over time did not differ significantly between the groups.

### Far Transfer

For the DSF and DSB (Wechsler, 2005), the OC group showed superior scores at T1 and T2 compared to the control group (see Table 2 and Figure 2B), but the main effect of group, was only significant for DSB, which reflects auditory working memory. DSF scores reflect short term auditory memory. The largest difference for DSB occurred between the groups after two full years of training.

The D2 test of attention (Brickenkamp and Zillmer, 1998) that measures selective and sustained attention and visual scanning speed (processing speed) showed a clear developmental trend for both groups, with a significant positive main effect of group, in favor of the OC children (see Table 2 and Figure 2B).

The MR scores (Wechsler, 2003) show a very similar development compared to the DSB scores, with a significant positive main effect of group for the OC group, with the most pronounced enhanced scores at T2.

For DSF, DSB, D2, and MR a progression in the scores showed between T1 and T2 in both groups. Interaction Time^∗^Group was not significant, which means that the all-over evolution of the scores over time did not differ significantly between the groups.

For the CCTT, results are less transparent (see Table 2 and Figure 2C). For both subtests (CCTT-1 measuring visual processing speed) and CCTT-2 (measuring visual processing speed and also cognitive flexibility), interaction Time^∗^Group was significant, whereas the overall main effect of group did not reach significance. We observed opposite trends in both groups: scores of the control group decreased from T1 to T2, but scores in the OC group increased. This is not due to baseline differences, as we controlled for T0 in the models (see Figure 1C).

To verify whether the observed progress in the scores between T1 and T2 for the OC group was significant, we applied Tukey corrected contrasts between the scores at T1 and T2 for CCTT-1 and CCTT-2 for both groups. For CCTT-1 no significant differences occurred in either group (OC group: *t* = −2.22, *p* = 0.129; control group: *t* = 1.45, *p* = 0.474). Tukey corrected contrasts for CCTT-2, however, confirmed significant progress between T1 and T2 for the OC group (*t* = −3.91, *p* = 0.012) but not for the control group (*t* = 0.85, *p* = 0.831).

For the three subtests of the Rey Auditory Verbal Learning Test, no significant differences exhibited between the groups (see Table 2 and Figure 2D). Only the second subtest (Rey-2), evaluating verbal learning showed a marginal main effect of group or “trend” with higher scores for the OC group (see Table 2). Verbal short-term memory (STM, Rey-1) and Verbal long-term memory (LTM, Rey-3) did not show any significant or marginal effects. Interaction Time^∗^Group was not significant for either of the three verbal tasks, therefore the evolution of the scores over time did not differ significantly between the groups.

### Sensorimotor Transfer

For all four subtests of the PP (Lafayette, 1999) the main effect of group was significant (see Table 2 and Figure 2E). However, for the simple peg inserting task with the right hand (PP_RH), the left hand (PP_LH) and with both hands (PP_BH), scores reached their summit already after 1 year of musical training in the OC group (at T1; see Figure 2E). For the more complex task, the assembly task (PP-Ass), scores increased gradually in both groups, and values were highest at T2. Interaction Time^∗^Group was not significant for either of the four sensorimotor tasks, thus, the evolution of the scores over time did not differ significantly between the groups.

## Discussion

This RCT compared practicing complex instruments to sensitization to music over the course of two full years in an intracurricular class setting in initially non-musician children in public primary schools.

We could show that after 2 years of intensive string instrument training, scores representing different musical, cognitive and sensorimotor functions in the OC group increased more than in the control group. This is all the more remarkable because the OC courses were not individual but taught within a complete class and on four different string instruments. Moreover, the control groups received the same amount of musical education, also in a full class setting. Teachers of both groups were professional musicians. We presume that learning to master a complex instrument, as well as the ensemble playing, (i.e., the dynamic interaction in the OC group, that requires the child to listen incessantly to the others and to adapt to the group and the teacher), constituted the driving force for this reinforced development.

We would like to emphasize that children would also mature and score better over time in the age groups we studied, without any musical interventions. This maturation derives partially from explicit learning within the regular school curriculum but also of from the spontaneous age-related acquisition of cognitive abilities in the context of natural child development (Siegler and Svetina, 2002). These superposed developmental trends may then be modulated by deliberate supplementary learning situations such as musical interventions and then differently so as a consequence of their nature (here focused instrumental training versus more dispersed sensitization to music). For this reason, we did not take the time variable separately into account, as progress in both groups would not add any relevant information with respect to the aim of our study: investigate the influence of two full years of intensive string instrument training in comparison to traditional music education on child development in a school setting.

Socioeconomic level and other background features of the child’s home situation also play a role in his/her level of involvement in musical activities in and outside the school setting. Personality traits of both parents and the child, as well as the child’s motivation and instrument preference, may strongly influence learning and thus the subsequent transfer to other cognitive and sensorimotor achievements (Hannon and Trainor, 2007; Southgate and Roscigno, 2009; Corrigall and Schellenberg, 2015). However, we consider that because the classes were assigned randomly to the OC and control programs in neighboring schools, and the study took place in public primary schools in popular low-income neighborhoods in the Geneva area, socioeconomic and other background features of the children were relatively balanced across the groups.

In the Geneva canton children are assigned to schools based on the neighborhood, which does not give the parents any choice. Therefore, it may be assumed that the socioeconomic level was more or less equally distributed in the different classes. Correcting for baseline performance, thus focusing on progress and not on initial differences, partially corrects for better performance at baseline for children from more privileged backgrounds. Excluding children who followed extracurricular music lessons may also have avoided certain advantages according to family provenance.

Finally, the baseline differences observed between the OC and the control group concerned AMMA-R and AMMA-C (the latter being a combination of both tonal and rhythmic scores so as to provide an overall musicality score) and for the PP assembly test measuring fine finger coordination between both hands. For these tests the control group performed better at T0 than the OC group (see Figure 1 and Supplementary Table 1). In particular, these three measures might be strongly influenced by string instrument training, Moreover, increased musical and complex sensorimotor functions at baseline might impact the performance of cognitive and sensorimotor tests at T1 and T2. We consider that we annihilated these preexisting differences by incorporating T0 into the models.

As we did in the “Results” section, we will discuss the effects in detail as a function of transfer.

### Close Transfer

The AMMA scores show stronger development of musical abilities that can be explained by a near transfer of learning in the OC group: Intensive musical instrumental training provokes more efficient processing of musical stimuli. Potential test–retest effects were in principle controlled for using an active control group but may play a role in the all-over increasing scores between T1 and T2 in both groups (this holds for all tests). However, we reshuffled the order of the melodies at each time point, and the melodies of this task are rather abstract and difficult to memorize after a year. This test can also be used repeatedly in adult professional musicians and does thus not easily show a ceiling effect (Gordon, 1989; Gordon, 2007). For the AMMA test, auditory working memory, attention and processing speed are crucial, as two subsequent melodies must be compared. This presumption is supported by the enhanced development of the DSB scores in the OC group. Moreover, development of visual attention and processing speed was also increased (D2 and CCTT-2 test). Assuming that visual and auditory attention and processing speed share common resources (Fougnie et al., 2018), this then constitutes a supplementary explanation for these near transfer results. Finally, cortical and subcortical functional and structural plasticity may also explain the observed advantages for processing music in the OC group (Trainor et al., 2009; Strait and Kraus, 2011; Zuk et al., 2014; Kraus and Strait, 2015; Tierney et al., 2015).

### Far Transfer

In addition, we observed far transfer of learning in several areas of general cognition. Far transfer of learning implies that the enhanced abilities extend beyond the boundaries of the trained domain, although there is little consensus on the precise nature of far transfer (Barnett and Ceci, 2002). According to Barnett and Ceci (2002), an important factor in defining far transfer is the spontaneity of its occurrence. Natural training procedures, based on real life experiences and not abstract manipulations, such as musical practice or dancing, are optimal for inducing generalized learning because they are complex and variable (Green and Bavelier, 2008; Green et al., 2013) and thus have a better chance of spontaneously inducing far transfer of learning.

In the current study, this transfer to other domains may be explained by the frequent use of certain core cognitive skills that are implicated in both musical practice and other cognitive functions (Bergman Nutley et al., 2014; Roden et al., 2014), such as working memory, attention, and processing speed. These basic cognitive abilities are strongly involved in and thus bolstered by musical training, and they may play a role as hubs, supporting more complex cognitive abilities like matrix reasoning (Cowan, 2014). So in our point of view, the transferred skills are not specific to music, but are rather general (Barnett and Ceci, 2002; Thaut, 2005), they are merely intensively trained during musical practice.

Working memory plays a more important role than short-term memory during music practice, as one has to continuously compare what just sounded with what is coming up, and this holds for the sounds produced by the player himself as well as for the surrounding musical context.

Concerning the CCTT scores, results were somewhat controversial, with higher scores, potentially learning effects, in the control group after 1 year, and recovery with superior scores after 2 years for the OC group. Notwithstanding, development from T1 to T2 manifested for both CCTT scores exclusively in the OC group, but only reached significance for CCTT-2. It is noteworthy that the reading of musical scores plays a much more important role during the second year of teaching of the OC program and may have impacted performing the CCTT-2 tasks stronger in the OC group in the second year, as score reading relies on visual scanning and processing speed, as well as cognitive flexibility. While reading the score the child has to adapt to a fluctuating auditory environment, especially in a group setting. Moreover, cognitive flexibility can be linked to enhanced sound discrimination (Saarikivi et al., 2016). In the first year, the children were rather focused on holding and handling their instrument, which is very difficult in the case of string instruments, as well as on basic audio-motor processing. In the second year, they could concentrate more on reading the score and listening to the others.

The absence of significant effects for the for the three subtests of the Rey Auditory Verbal Learning Test may surprise some, as the increase of verbal memory and other language functions is often reported as an effect of musical training (Ho et al., 2003; Roden et al., 2012; Jaschke et al., 2018b). However, we should acknowledge that (1) the control group was also musically trained, and sensitization to music did show a trend for verbal IQ (Jaschke et al., 2018a); (2) children in which verbal advantages were observed started musical training at a younger age on average than the groups tested in the current experiment (Ho et al., 2003; Roden et al., 2012; Jaschke et al., 2018b); and (3) that we did find a marginal positive effect of verbal learning in the OC group as compared to the control group. As our groups were of medium size this may have prevented us from reaching statistical significance for this variable following a lack of statistical power.

### Sensorimotor Transfer

We would classify the sensorimotor transfer as intermediate transfer (i.e., in between close and far transfer). Playing a string instrument, which demands a very asymmetric right-versus-left motor coordination, versus inserting or assembling small metal objects in the frontal plane, as required in our sensorimotor test (PP), are not that closely related, although both require manual dexterity and bimanual coordination.

In all four subtests, the OC group outperformed the control group. The impact of the musical instrumental practice on sensorimotor performance was most obvious after the first year of training for the three simple peg inserting tasks (PP-RH, PP-LH, PP-BH; see the section “Purdue Pegboard”). This seems plausible, as learning to hold and handle a string instrument, involving the right and the left hand in very different ways is quite demanding in the beginning. In particular, the fine dexterity of the fingers of the left hand is particularly challenging. Concordantly, the effect size of the PP-LH and PP-BH tasks was larger than for the right hand (PP-RH; see Table 2). For the more complex task, the assembly task (PP-Ass), demanding fine finger dexterity and advanced bimanual coordination, scores increased gradually in both groups, and values were highest at T2. The control group manifested the same pattern over time, but with lower scores on average than the OC group.

## Conclusion

The merit of the study presented here is that two groups of initially musically naïve children were compared for two different musical group interventions: focused instrumental training and sensitization to music, both as part of the normal school curriculum.

We could show that learning to play a complex instrument in a group setting for over 2 years, positively impacts general cognitive and sensorimotor behavior much stronger than sensitization to music, even if the latter also comprises some musical practice. Our results therefore highlight the added value of intensive musical instrumental training in a group setting, encouraging its general implementation in public primary schools.

Core functions such as working memory, attention, processing speed, and cognitive flexibility, as well as hand dexterity, bimanual coordination and also abstract thinking, were enhanced in the OC group as compared to the sensitization to music group after 2 years of musical training.

These data show that intensive practice of a complex musical instrument associated with ensemble playing is a powerful means to enhance the development of core cognitive and executive functions of the primary school child and thus better preparing him/her for secondary education. Executive functions and abstract reasoning most likely support academic achievement most strongly (Cortés Pascual et al., 2019). Just being sensitized to music is not sufficient to bring about such changes.

The motivational and emotional aspects of musical practice could also be an explanation for the facilitation of learning (Ferreri and Verga, 2016). Making or appreciating music affects the dopaminergic and other hormone and endocrine systems (Blood and Zatorre, 2001; Chanda and Levitin, 2013; Ferreri et al., 2019) and could reinforce learning. Given the fact that the children were playing together, and together with the teachers, and that the chosen musical material was child friendly and stimulating, enhanced dopamine release is probable.

One may wonder whether the observed benefits will remain stable over time. However, the positive influence on general intelligence (IQ) of musical practice compared to other artistic activities appeared to be sustainable over time (Schellenberg, 2006). The authors concluded that practicing music during childhood provokes a moderate but lasting positive effect on intelligence and academic performance. A recent study of twins showed that playing a musical instrument in their younger years, taking into account gender, education and physical activity, reduces the risk of dementia and cognitive impairment in old age (Balbag et al., 2014), and the same was found in other unrelated elderly individuals (Hanna-Pladdy and Mackay, 2011; White-Schwoch et al., 2013).

### Limitations of the Study

The age group studied here (10–12 years) is not ideal to show optimal benefits of musical practice and training. Neuronal plasticity is at its peak at around 7 years of age (Wan and Schlaug, 2010). An earlier start as well as a longer period of training could provoke stronger enhancement of development. The observed developmental enhancements in the OC group are nevertheless considerable. On the local political level of the Geneva canton, the results generated by this study provoked a prolongation of the OC program, which will now start 2 years earlier and last 4 years instead of two. Nonetheless, starting music practice in adolescence (Tierney et al., 2015) or even in old age (Bugos et al., 2007; Seinfeld et al., 2013; Dege and Kerkovius, 2018), can still provoke benefits, as our brains are plastic from the cradle to the grave.

Although the control group allowed us to verify for test–retest learning effects we cannot exclude that some of the progress in both groups was partially supported by such learning effects.

## Data Availability Statement

The datasets generated/analyzed for this study can be found in the yareta repository (https://yareta.unige.ch; 10.26037/yareta:dhglarpzdfcgzefx4pky7sua3m), a FAIR digital solution for long-term preservation and sharing of research data developed by the University of Geneva under the national project swissuniversities DLCM (https://www.dlcm.ch/) of which the HES-SO is a partner.

## Ethics Statement

The studies involving human participants were reviewed and approved by Commission d’éthique de la Faculté de Psychologie et des Sciences de l’Education de l’Université de Genève. Written informed consent to participate in this study was provided by the participants’ legal guardian/next of kin.

## Author Contributions

CJ, SZ, and MK conceived and designed the experiments. LA and DG passed the tests and organized the data. CJ and ED-L analyzed the data. CJ, MK, SZ, and ED-L wrote the manuscript.

## Conflict of Interest

The authors declare that the research was conducted in the absence of any commercial or financial relationships that could be construed as a potential conflict of interest.

## Footnotes

1 https://www.giamusic.com/products/P-3372.cfm

2 https://CRAN.R-project.org/package=emmeans
