# Supplementary material for: Formal String Instrument Training in a Class Setting Enhances Cognitive and Sensorimotor Development of Primary School Children
Source: Front Neurosci. 2020 Jun 16;14:567. doi: 10.3389/fnins.2020.00567 (PMC7309442; doi:10.3389/fnins.2020.00567)
Supplement: Supplementary file 1 [file Table_1.docx]

**Supplementary Table 1:** Descriptive Values by Group, with added p-values at T0 comparing the OC group and the control group in column four (OC vs control [Ctrl]), evaluated by means of t-tests for continuous variables and with chi-square tests for categorical variables. Significant effects are represented in bold font. Marginal but non-significant effects (p = 0.05-0.1) are represented in italic font.

|  | Control (N=35) | OC (N=34) | p-value (OC vs Ctrl) | Total (N=69) |
| --- | --- | --- | --- | --- |
| **SEX** |  |  | 0.378 |  |
| F | 19 (54.3%) | 22 (64.7%) |  | 41 (59.4%) |
| M | 16 (45.7%) | 12 (35.3%) |  | 28 (40.6%) |
| Missing | 0 | 0 |  | 0 |
| **Age_2016** |  |  | 0.176 |  |
| Mean (SD) | 10.22 (0.34) | 10.12 (0.27) |  | 10.17 (0.31) |
| Median (Q1, Q3) | 10.18 (9.98, 10.46) | 10.09 (9.91, 10.35) |  | 10.14 (9.92, 10.43) |
| Min - Max | 9.76 - 11.19 | 9.70 - 10.55 |  | 9.70 - 11.19 |
| Missing | 1 | 1 |  | 2 |
| **Handedness** |  |  | 0.660 |  |
| Right | 32 (91.4%) | 30 (88.2%) |  | 62 (89.9%) |
| Left | 3 (8.6%) | 4 (11.8%) |  | 7 (10.1%) |
| Missing | 0 | 0 |  | 0 |
| **AMMA-T_T0** |  |  | *0.062* |  |
| Mean (SD) | 36.21 (20.51) | 27.15 (18.80) |  | 31.68 (20.05) |
| Median (Q1, Q3) | 39.00 (24.00, 53.75) | 24.00 (10.00, 39.00) |  | 29.00 (14.00, 44.00) |
| Min - Max | 3.00 - 88.00 | 5.00 - 88.00 |  | 3.00 - 88.00 |
| Missing | 1 | 0 |  | 1 |
| **AMMA-T_T1** |  |  |  |  |
| Mean (SD) | 35.41 (20.57) | 41.27 (26.31) |  | 38.30 (23.58) |
| Median (Q1, Q3) | 39.00 (20.25, 50.00) | 34.00 (24.00, 57.00) |  | 39.00 (21.50, 57.00) |
| Min - Max | 2.00 - 69.00 | 4.00 - 91.00 |  | 2.00 - 91.00 |
| Missing | 1 | 1 |  | 2 |
| **AMMA-T_T2** |  |  |  |  |
| Mean (SD) | 38.91 (25.45) | 50.23 (28.32) |  | 44.48 (27.28) |
| Median (Q1, Q3) | 39.00 (14.00, 57.00) | 50.00 (24.00, 74.00) |  | 39.00 (24.00, 66.00) |
| Min - Max | 5.00 - 88.00 | 5.00 - 99.00 |  | 5.00 - 99.00 |
| Missing | 3 | 3 |  | 6 |
| **AMMA-R_T0** |  |  | **0.046** |  |
| Mean (SD) | 27.06 (18.38) | 18.62 (15.82) |  | 22.84 (17.54) |
| Median (Q1, Q3) | 27.50 (11.00, 35.00) | 15.00 (4.00, 30.00) |  | 25.00 (7.00, 31.25) |
| Min - Max | 1.00 - 85.00 | 1.00 - 56.00 |  | 1.00 - 85.00 |
| Missing | 1 | 0 |  | 1 |
| **AMMA-R_T1** |  |  |  |  |
| Mean (SD) | 23.35 (18.70) | 32.03 (21.97) |  | 27.63 (20.69) |
| Median (Q1, Q3) | 20.00 (11.00, 30.00) | 30.00 (20.00, 40.00) |  | 25.00 (11.00, 40.00) |
| Min - Max | 1.00 - 74.00 | 1.00 - 89.00 |  | 1.00 - 89.00 |
| Missing | 1 | 1 |  | 2 |
| **AMMA-R_T2** |  |  |  |  |
| Mean (SD) | 29.84 (24.34) | 38.39 (27.30) |  | 34.05 (25.99) |
| Median (Q1, Q3) | 25.00 (11.00, 40.00) | 40.00 (17.50, 56.00) |  | 25.00 (11.00, 56.00) |
| Min - Max | 1.00 - 80.00 | 1.00 - 93.00 |  | 1.00 - 93.00 |
| Missing | 3 | 3 |  | 6 |
| **AMMA-C_T0** |  |  | **0.025** |  |
| Mean (SD) | 28.76 (17.52) | 19.76 (14.61) |  | 24.26 (16.64) |
| Median (Q1, Q3) | 27.50 (15.00, 38.00) | 17.00 (7.25, 29.00) |  | 23.00 (10.00, 35.00) |
| Min - Max | 2.00 - 74.00 | 4.00 - 68.00 |  | 2.00 - 74.00 |
| Missing | 1 | 0 |  | 1 |
| **AMMA-C_T1** |  |  |  |  |
| Mean (SD) | 25.38 (16.45) | 34.03 (22.07) |  | 29.64 (19.76) |
| Median (Q1, Q3) | 29.00 (10.50, 34.25) | 29.00 (23.00, 38.00) |  | 29.00 (14.00, 38.00) |
| Min - Max | 2.00 - 62.00 | 4.00 - 89.00 |  | 2.00 - 89.00 |
| Missing | 1 | 1 |  | 2 |
| **AMMA-C_T2** |  |  |  |  |
| Mean (SD) | 31.84 (24.04) | 43.68 (27.93) |  | 37.67 (26.50) |
| Median (Q1, Q3) | 26.00 (13.00, 41.75) | 47.00 (17.00, 69.50) |  | 32.00 (15.00, 58.00) |
| Min - Max | 5.00 - 84.00 | 1.00 - 88.00 |  | 1.00 - 88.00 |
| Missing | 3 | 3 |  | 6 |
| **DSF_T0** |  |  | 0.879 |  |
| Mean (SD) | 7.46 (2.16) | 7.53 (1.73) |  | 7.49 (1.95) |
| Median (Q1, Q3) | 7.00 (6.00, 8.00) | 7.00 (6.00, 8.75) |  | 7.00 (6.00, 8.00) |
| Min - Max | 4.00 - 15.00 | 5.00 - 12.00 |  | 4.00 - 15.00 |
| Missing | 0 | 0 |  | 0 |
| **DSF_T1** |  |  |  |  |
| Mean (SD) | 7.82 (1.90) | 8.15 (1.73) |  | 7.99 (1.81) |
| Median (Q1, Q3) | 8.00 (7.00, 8.00) | 8.00 (7.00, 9.00) |  | 8.00 (7.00, 9.00) |
| Min - Max | 5.00 - 14.00 | 6.00 - 12.00 |  | 5.00 - 14.00 |
| Missing | 1 | 1 |  | 2 |
| **DSF_T2** |  |  |  |  |
| Mean (SD) | 8.12 (2.03) | 8.41 (1.85) |  | 8.27 (1.93) |
| Median (Q1, Q3) | 8.00 (6.75, 9.00) | 8.00 (7.00, 9.00) |  | 8.00 (7.00, 9.00) |
| Min - Max | 5.00 - 14.00 | 6.00 - 13.00 |  | 5.00 - 14.00 |
| Missing | 3 | 2 |  | 5 |
| **DSB_T0** |  |  | 0.148 |  |
| Mean (SD) | 6.77 (1.44) | 6.26 (1.44) |  | 6.52 (1.45) |
| Median (Q1, Q3) | 6.00 (6.00, 8.00) | 6.00 (5.00, 7.00) |  | 6.00 (5.00, 7.00) |
| Min - Max | 5.00 - 10.00 | 4.00 - 10.00 |  | 4.00 - 10.00 |
| Missing | 0 | 0 |  | 0 |
| **DSB_T1** |  |  |  |  |
| Mean (SD) | 7.12 (1.68) | 7.00 (1.79) |  | 7.06 (1.72) |
| Median (Q1, Q3) | 7.00 (6.25, 8.00) | 7.00 (6.00, 8.00) |  | 7.00 (6.00, 8.00) |
| Min - Max | 4.00 - 14.00 | 4.00 - 11.00 |  | 4.00 - 14.00 |
| Missing | 1 | 1 |  | 2 |
| **DSB_T2** |  |  |  |  |
| Mean (SD) | 7.25 (1.80) | 7.91 (1.69) |  | 7.58 (1.76) |
| Median (Q1, Q3) | 7.00 (6.00, 8.00) | 8.00 (6.75, 9.00) |  | 8.00 (6.00, 9.00) |
| Min - Max | 5.00 - 12.00 | 4.00 - 11.00 |  | 4.00 - 12.00 |
| Missing | 3 | 2 |  | 5 |
| **D2_T0** |  |  | 0.422 |  |
| Mean (SD) | 314.29 (61.57) | 303.26 (51.06) |  | 308.86 (56.49) |
| Median (Q1, Q3) | 315.00 (277.50, 342.50) | 304.00 (273.50, 323.75) |  | 307.00 (272.00, 341.00) |
| Min - Max | 199.00 - 521.00 | 175.00 - 406.00 |  | 175.00 - 521.00 |
| Missing | 0 | 0 |  | 0 |
| **D2_T1** |  |  |  |  |
| Mean (SD) | 373.56 (47.46) | 379.76 (47.91) |  | 376.61 (47.42) |
| Median (Q1, Q3) | 371.50 (344.50, 410.75) | 374.00 (335.00, 420.00) |  | 374.00 (342.00, 413.00) |
| Min - Max | 276.00 - 487.00 | 290.00 - 462.00 |  | 276.00 - 487.00 |
| Missing | 1 | 1 |  | 2 |
| **D2_T2** |  |  |  |  |
| Mean (SD) | 412.09 (51.29) | 427.41 (53.71) |  | 419.75 (52.66) |
| Median (Q1, Q3) | 419.00 (378.75, 450.75) | 421.00 (387.75, 463.25) |  | 419.00 (386.75, 459.75) |
| Min - Max | 299.00 - 495.00 | 331.00 - 552.00 |  | 299.00 - 552.00 |
| Missing | 3 | 2 |  | 5 |
| **MR_T0** |  |  | 0.103 |  |
| Mean (SD) | 20.74 (3.58) | 19.09 (4.67) |  | 19.93 (4.21) |
| Median (Q1, Q3) | 21.00 (19.00, 23.00) | 20.00 (15.25, 22.00) |  | 21.00 (17.00, 23.00) |
| Min - Max | 11.00 - 26.00 | 11.00 - 27.00 |  | 11.00 - 27.00 |
| Missing | 0 | 0 |  | 0 |
| **MR_T1** |  |  |  |  |
| Mean (SD) | 23.94 (3.09) | 23.85 (3.37) |  | 23.90 (3.21) |
| Median (Q1, Q3) | 24.00 (23.00, 26.00) | 23.00 (22.00, 26.00) |  | 24.00 (22.00, 26.00) |
| Min - Max | 15.00 - 29.00 | 16.00 - 33.00 |  | 15.00 - 33.00 |
| Missing | 1 | 1 |  | 2 |
| **MR_T2** |  |  |  |  |
| Mean (SD) | 24.34 (3.58) | 25.91 (2.97) |  | 25.12 (3.35) |
| Median (Q1, Q3) | 23.50 (22.00, 27.00) | 26.00 (23.75, 28.00) |  | 25.00 (22.00, 28.00) |
| Min - Max | 18.00 - 31.00 | 21.00 - 32.00 |  | 18.00 - 32.00 |
| Missing | 3 | 2 |  | 5 |
| **CCTT-1_T0** |  |  | 0.767 |  |
| Mean (SD) | 29.86 (29.52) | 31.85 (26.02) |  | 30.84 (27.66) |
| Median (Q1, Q3) | 18.00 (4.50, 50.00) | 27.00 (10.50, 49.00) |  | 21.00 (8.00, 50.00) |
| Min - Max | 1.00 - 96.00 | 1.00 - 84.00 |  | 1.00 - 96.00 |
| Missing | 0 | 0 |  | 0 |
| **CCTT-1_T1** |  |  |  |  |
| Mean (SD) | 50.26 (28.59) | 41.15 (27.27) |  | 45.78 (28.11) |
| Median (Q1, Q3) | 66.00 (21.00, 73.00) | 46.00 (14.00, 58.00) |  | 54.00 (18.00, 71.00) |
| Min - Max | 1.00 - 82.00 | 1.00 - 92.00 |  | 1.00 - 92.00 |
| Missing | 1 | 1 |  | 2 |
| **CCTT-1_T2** |  |  |  |  |
| Mean (SD) | 40.97 (23.80) | 55.53 (27.53) |  | 48.25 (26.56) |
| Median (Q1, Q3) | 40.00 (18.00, 58.00) | 66.00 (37.00, 79.00) |  | 46.00 (25.50, 70.00) |
| Min - Max | 2.00 - 84.00 | 1.00 - 90.00 |  | 1.00 - 90.00 |
| Missing | 3 | 2 |  | 5 |
| **CCTT-2_T0** |  |  | 0.777 |  |
| Mean (SD) | 42.94 (23.05) | 41.36 (22.69) |  | 42.18 (22.72) |
| Median (Q1, Q3) | 38.00 (24.00, 64.00) | 42.00 (21.00, 58.00) |  | 42.00 (21.00, 62.00) |
| Min - Max | 1.00 - 82.00 | 4.00 - 79.00 |  | 1.00 - 82.00 |
| Missing | 0 | 1 |  | 1 |
| **CCTT-2_T1** |  |  |  |  |
| Mean (SD) | 55.53 (20.74) | 42.82 (23.46) |  | 49.27 (22.87) |
| Median (Q1, Q3) | 58.00 (46.00, 69.00) | 42.00 (24.00, 66.00) |  | 54.00 (31.00, 66.00) |
| Min - Max | 1.00 - 90.00 | 3.00 - 86.00 |  | 1.00 - 90.00 |
| Missing | 1 | 1 |  | 2 |
| **CCTT-2_T2** |  |  |  |  |
| Mean (SD) | 51.06 (25.51) | 58.25 (20.32) |  | 54.66 (23.16) |
| Median (Q1, Q3) | 62.00 (26.25, 73.00) | 60.00 (50.00, 73.00) |  | 60.00 (37.00, 73.00) |
| Min - Max | 2.00 - 82.00 | 12.00 - 88.00 |  | 2.00 - 88.00 |
| Missing | 3 | 2 |  | 5 |
| **Rey-1_T0** |  |  | 0.313 |  |
| Mean (SD) | 6.77 (1.94) | 6.29 (1.96) |  | 6.54 (1.95) |
| Median (Q1, Q3) | 6.00 (6.00, 7.50) | 6.00 (4.25, 7.00) |  | 6.00 (5.00, 7.00) |
| Min - Max | 4.00 - 11.00 | 3.00 - 10.00 |  | 3.00 - 11.00 |
| Missing | 0 | 0 |  | 0 |
| **Rey-1_T1** |  |  |  |  |
| Mean (SD) | 6.21 (1.41) | 6.18 (1.65) |  | 6.19 (1.52) |
| Median (Q1, Q3) | 6.00 (5.00, 7.00) | 6.00 (5.00, 7.00) |  | 6.00 (5.00, 7.00) |
| Min - Max | 4.00 - 10.00 | 2.00 - 10.00 |  | 2.00 - 10.00 |
| Missing | 1 | 1 |  | 2 |
| **Rey-1_T2** |  |  |  |  |
| Mean (SD) | 6.78 (1.56) | 6.44 (1.74) |  | 6.61 (1.65) |
| Median (Q1, Q3) | 6.50 (5.75, 8.00) | 6.00 (5.00, 8.00) |  | 6.00 (5.00, 8.00) |
| Min - Max | 4.00 - 10.00 | 3.00 - 10.00 |  | 3.00 - 10.00 |
| Missing | 3 | 2 |  | 5 |
| **Rey-2_T0** |  |  | 0.486 |  |
| Mean (SD) | 11.21 (2.04) | 11.51 (1.51) |  | 11.36 (1.79) |
| Median (Q1, Q3) | 11.20 (10.25, 12.47) | 11.70 (10.62, 12.50) |  | 11.50 (10.50, 12.50) |
| Min - Max | 6.75 - 15.30 | 7.25 - 14.00 |  | 6.75 - 15.30 |
| Missing | 0 | 0 |  | 0 |
| **Rey-2_T1** |  |  |  |  |
| Mean (SD) | 11.62 (1.50) | 12.00 (1.26) |  | 11.81 (1.39) |
| Median (Q1, Q3) | 12.00 (10.81, 12.75) | 12.25 (11.75, 12.50) |  | 12.00 (11.12, 12.75) |
| Min - Max | 8.50 - 13.75 | 8.50 - 14.50 |  | 8.50 - 14.50 |
| Missing | 1 | 1 |  | 2 |
| **Rey-2_T2** |  |  |  |  |
| Mean (SD) | 11.47 (1.61) | 12.09 (1.43) |  | 11.78 (1.54) |
| Median (Q1, Q3) | 11.50 (10.25, 12.75) | 12.25 (11.44, 13.06) |  | 12.00 (10.88, 13.00) |
| Min - Max | 8.50 - 14.00 | 8.50 - 14.50 |  | 8.50 - 14.50 |
| Missing | 3 | 2 |  | 5 |
| **Rey-3_T0** |  |  | 0.408 |  |
| Mean (SD) | 12.46 (2.03) | 12.85 (1.91) |  | 12.65 (1.97) |
| Median (Q1, Q3) | 12.00 (12.00, 14.00) | 13.00 (12.00, 14.00) |  | 13.00 (12.00, 14.00) |
| Min - Max | 5.00 - 15.00 | 7.00 - 15.00 |  | 5.00 - 15.00 |
| Missing | 0 | 0 |  | 0 |
| **Rey-3_T1** |  |  |  |  |
| Mean (SD) | 13.04 (1.79) | 13.09 (2.24) |  | 13.06 (2.01) |
| Median (Q1, Q3) | 13.00 (12.00, 15.00) | 14.00 (12.00, 15.00) |  | 14.00 (12.00, 15.00) |
| Min - Max | 9.00 - 15.00 | 8.00 - 15.00 |  | 8.00 - 15.00 |
| Missing | 1 | 1 |  | 2 |
| **Rey-3_T2** |  |  |  |  |
| Mean (SD) | 11.34 (2.40) | 12.25 (2.00) |  | 11.80 (2.24) |
| Median (Q1, Q3) | 11.00 (9.75, 14.00) | 12.00 (11.00, 13.25) |  | 12.00 (11.00, 14.00) |
| Min - Max | 6.00 - 14.00 | 7.00 - 15.00 |  | 6.00 - 15.00 |
| Missing | 3 | 2 |  | 5 |
| **P-RH_T0** |  |  | 0.154 |  |
| Mean (SD) | 12.69 (2.68) | 11.85 (2.08) |  | 12.28 (2.42) |
| Median (Q1, Q3) | 13.00 (11.00, 14.00) | 12.00 (11.00, 13.75) |  | 12.00 (11.00, 14.00) |
| Min - Max | 8.00 - 19.00 | 7.00 - 15.00 |  | 7.00 - 19.00 |
| Missing | 0 | 0 |  | 0 |
| **P-RH_T1** |  |  |  |  |
| Mean (SD) | 15.44 (1.60) | 15.91 (1.55) |  | 15.67 (1.58) |
| Median (Q1, Q3) | 16.00 (14.25, 16.00) | 16.00 (15.00, 17.00) |  | 16.00 (15.00, 16.00) |
| Min - Max | 13.00 - 19.00 | 12.00 - 19.00 |  | 12.00 - 19.00 |
| Missing | 1 | 1 |  | 2 |
| **P-RH_T2** |  |  |  |  |
| Mean (SD) | 14.47 (1.68) | 15.22 (1.81) |  | 14.84 (1.77) |
| Median (Q1, Q3) | 14.50 (13.00, 16.00) | 15.00 (14.00, 16.00) |  | 15.00 (14.00, 16.00) |
| Min - Max | 11.00 - 19.00 | 12.00 - 21.00 |  | 11.00 - 21.00 |
| Missing | 3 | 2 |  | 5 |
| **P-LH_T0** |  |  | 0.818 |  |
| Mean (SD) | 12.74 (2.14) | 12.62 (2.06) |  | 12.68 (2.08) |
| Median (Q1, Q3) | 12.50 (11.00, 15.00) | 12.50 (11.25, 14.00) |  | 12.50 (11.00, 15.00) |
| Min - Max | 8.00 - 16.00 | 8.00 - 16.00 |  | 8.00 - 16.00 |
| Missing | 1 | 0 |  | 1 |
| **P-LH_T1** |  |  |  |  |
| Mean (SD) | 13.09 (1.85) | 14.30 (1.83) |  | 13.69 (1.92) |
| Median (Q1, Q3) | 13.00 (12.00, 14.00) | 15.00 (13.00, 15.00) |  | 13.00 (12.00, 15.00) |
| Min - Max | 9.00 - 17.00 | 11.00 - 18.00 |  | 9.00 - 18.00 |
| Missing | 1 | 1 |  | 2 |
| **P-LH_T2** |  |  |  |  |
| Mean (SD) | 12.75 (1.74) | 13.56 (1.64) |  | 13.16 (1.73) |
| Median (Q1, Q3) | 13.00 (11.75, 13.25) | 14.00 (12.75, 14.25) |  | 13.00 (12.00, 14.00) |
| Min - Max | 10.00 - 18.00 | 10.00 - 17.00 |  | 10.00 - 18.00 |
| Missing | 3 | 2 |  | 5 |
| **P-BH_T0** |  |  | 0.172 |  |
| Mean (SD) | 17.50 (5.70) | 15.50 (6.25) |  | 16.50 (6.02) |
| Median (Q1, Q3) | 20.00 (11.25, 22.00) | 12.50 (10.00, 22.00) |  | 18.00 (10.00, 22.00) |
| Min - Max | 8.00 - 28.00 | 6.00 - 26.00 |  | 6.00 - 28.00 |
| Missing | 1 | 0 |  | 1 |
| **P-BH_T1** |  |  |  |  |
| Mean (SD) | 22.94 (3.05) | 24.42 (3.49) |  | 23.67 (3.34) |
| Median (Q1, Q3) | 24.00 (20.00, 24.75) | 24.00 (22.00, 26.00) |  | 24.00 (22.00, 26.00) |
| Min - Max | 18.00 - 30.00 | 16.00 - 32.00 |  | 16.00 - 32.00 |
| Missing | 1 | 1 |  | 2 |
| **P-BH_T2** |  |  |  |  |
| Mean (SD) | 21.66 (3.62) | 23.19 (3.51) |  | 22.42 (3.62) |
| Median (Q1, Q3) | 22.00 (19.50, 24.00) | 24.00 (22.00, 26.00) |  | 22.50 (20.00, 26.00) |
| Min - Max | 16.00 - 30.00 | 16.00 - 28.00 |  | 16.00 - 30.00 |
| Missing | 3 | 2 |  | 5 |
| **P-Ass_T0** |  |  | **0.046** |  |
| Mean (SD) | 28.29 (7.65) | 23.44 (11.62) |  | 25.87 (10.06) |
| Median (Q1, Q3) | 30.50 (25.00, 33.00) | 25.50 (9.25, 33.00) |  | 30.00 (21.50, 33.00) |
| Min - Max | 7.00 - 38.00 | 6.00 - 40.00 |  | 6.00 - 40.00 |
| Missing | 1 | 0 |  | 1 |
| **P-Ass_T1** |  |  |  |  |
| Mean (SD) | 34.09 (5.75) | 36.36 (4.05) |  | 35.21 (5.08) |
| Median (Q1, Q3) | 33.50 (30.00, 38.75) | 36.00 (34.00, 38.00) |  | 36.00 (32.00, 38.50) |
| Min - Max | 20.00 - 44.00 | 26.00 - 44.00 |  | 20.00 - 44.00 |
| Missing | 1 | 1 |  | 2 |
| **P-Ass_T2** |  |  |  |  |
| Mean (SD) | 35.62 (4.51) | 36.66 (5.36) |  | 36.14 (4.94) |
| Median (Q1, Q3) | 36.00 (33.75, 38.25) | 38.00 (32.00, 40.00) |  | 36.00 (33.00, 40.00) |
| Min - Max | 25.00 - 46.00 | 24.00 - 46.00 |  | 24.00 - 46.00 |
| Missing | 3 | 2 |  | 5 |
